# Prevalence of disordered eating behaviors in adolescents with type 1 diabetes: Results of multicenter Italian nationwide study

**DOI:** 10.1002/eat.23764

**Published:** 2022-06-25

**Authors:** Alda Troncone, Gaetana Affuso, Crescenzo Cascella, Antonietta Chianese, Barbara Pizzini, Angela Zanfardino, Dario Iafusco, Diabetes Study Group of Italian Society of Paediatric Endocrinology and Diabetology

**Affiliations:** ^1^ Department of Psychology University of Campania “Luigi Vanvitelli” Caserta Italy; ^2^ Department of the Woman, of the Child and of the General and Specialized Surgery University of Campania “Luigi Vanvitelli” Naples Italy

**Keywords:** adolescence, disordered eating behaviors, emotional and behavioral problems, type 1 diabetes

## Abstract

**Objective:**

To assess the prevalence of disordered eating behaviors (DEBs) in a large sample of Italian adolescents with type 1 diabetes and to explore potential demographic, clinical, and psychological differences (understood as emotional and behavioral problems) among adolescents with and without DEBs.

**Method:**

Adolescents (11–19 years) with type 1 diabetes completed the Diabetes Eating Problems Survey‐revised (DEPS‐r) and the Youth Self Report (YSR). Demographic and clinical data were also collected.

**Results:**

Of 690 adolescents with type 1 diabetes (mean age 14.97 ± 1.81, *n* = 337 girls) assessed in this study, 28.1% (21% boys, 35% girls) were DEPS‐r positive (score ≥ 20). Girls had higher DEPS‐r total scores (*p* < .0001, *d* = .42) than boys, although no age differences were found in mean DEPS‐r total scores (*p* = .961). In both genders, adolescents with DEBs had significantly higher zBMI (*p* < .0001, *d* = .52) and HbA1c values (*p* < .0001, *d* = .54) and showed more emotional and behavioral problems (both as internalizing and externalizing problems) than those without DEBs (all *p* < .0001). These differences were largely confirmed in all age groups. Adolescents reporting insulin misuse had higher HbA1c values (*p* = .001, *d* = .26), higher DEPS‐r mean scores (*p* < .0001, *d* = 1.07), and greater psychological problems (all *p* < .001) than those who did not.

**Discussion:**

DEBs are prevalent among adolescents with type 1 diabetes, and those with eating problems showed adverse clinical and psychological conditions. Routine screening for DEBs and of general psychological condition should be a fundamental part of diabetes care, especially during adolescence.

**Public significance statement:**

This nationwide study indicated that DEBs are common in adolescents with T1D, and those suffering from them show poorer clinical conditions and higher emotional and behavioral problems. As such, it offers important contributions for those working with EDs and in the T1D field, as it provides a deeper understanding of the co‐occurring DEBs–emotional/behavioral problems in youths with T1D and highlights the importance of continuous monitoring of their psychological condition by a multidisciplinary team.

## 1 INTRODUCTION

Type 1 diabetes (T1D) is an immune‐mediated disorder characterized by hyperglycemia resulting from a deficit or absence of endogenous insulin due to the destruction of pancreas beta cells. T1D management is a psychologically complex process that requires adhering to a demanding structured plan, including a prescribed pharmacological regime (e.g., multiple daily insulin therapy), regular blood glucose checking, proper nutritional management (e.g., monitoring carbohydrate intake), and regular physical activity (Chiang et al., 2018). Achieving adequate metabolic control—that is, maintaining one's blood glucose at a level as close as possible to the physiological range—to reduce future complications is the primary goal of diabetes therapy.

There is a general consensus that individuals with T1D are at increased risk of developing disordered eating behaviors (DEBs) (i.e., abnormal eating attitudes and behaviors, including unhealthy weight‐control practices, binge eating, chronic dieting, vomiting, overeating, breaking dietary rules, etc.) (Broadley et al., 2020; Conviser et al., 2018). It is believed that specific factors of daily T1D management (e.g., a continuous focus on food and eating, dietary restraints, eating to treat low‐blood glucose, insulin‐related weight gain, etc.) may play an iatrogenic role (Philpot, 2013; Pinhas‐Hamiel et al., 2015).

As in the healthy general population, adolescence is also considered a risk factor for individuals with T1D to develop DEBs (Hanlan et al., 2013). In particular, in terms of prevalence of co‐occurring T1D–DEBs, reviews have estimated the overall mean of DEBs in adolescents with T1D at 39.3% (vs. 32.5% in those without T1D) (Pinhas‐Hamiel et al., 2015; Young et al., 2013). Overall, DEB estimation rates in adolescents with T1D vary across studies, with regard to the basis of definitions and to the methods not always including diabetes‐specific measures (Pursey et al., 2020).

It should be noted that although the prevalence of DEBs in adolescents with T1D has frequently been investigated (e.g., Ackard et al., 2008; Colton et al., 2015; Nilsson et al., 2020), few nationally representative epidemiological/cohort studies have been conducted. To date, nationwide population‐based studies have investigated DEB occurrence in Norwegian (*N* = 770) (Wisting et al., 2013a), German/Austrian (*N* = 380) (Scheuing et al., 2014), German (*N* = 819) (Bächle et al., 2016), Australian (*N* = 477) (Araia et al., 2017), and US (*N* = 2156) (Nip et al., 2019) pediatric and young adult patients with T1D. In Italy, the DEB prevalence in adolescents with T1D has only been estimated in small, convenience‐based samples (Calcaterra et al., 2020; Cherubini et al., 2018; Troncone et al., 2019; Troncone, Cascella, Chianese, Zanfardino, Piscopo, et al., 2020; Troncone, Chianese, Zanfardino, Cascella, Piscopo et al., 2020a).

Even though studies in individuals without T1D (Keski‐Rahkonen & Mustelin, 2016; Swanson et al., 2011) have shown that eating problems are strongly associated with psychopathological conditions (e.g., mood and anxiety disorders), in most studies analyzing the co‐occurrence of T1D and DEBs in adolescents, associated psychopathological comorbidities have received relatively little attention. To date, the few studies that have assessed comorbid psychopathology in adolescents with T1D and DEBs described the latter as being associated with executive function problems (Cecilia‐Costa et al., 2021), self‐regulation deficits (Grylli et al., 2010), emotion regulation strategies difficulties (Yilmaz Kafali et al., 2020), and emotional and behavioral problems (d'Emden et al., 2013; Troncone et al., 2020).

The worldwide estimate of the number of children and adolescents with T1D continues to increase dramatically (it is estimated that 600,900 children under 15 years old live with T1D worldwide and 98,200 children under 15 years old develop T1D annually; Patterson et al., 2019); thus, identifying the prevalence, severity, and characteristics of co‐occurring psychological problems is crucial to making advance in general diabetes psychological care. Specifically, given the positive association in adolescents with T1D between psychopathology and poor metabolic control (Northam et al., 2005), as well as the increased risk of diabetes‐related complications and mortality associated with co‐occurring T1D–DEBs (Goebel‐Fabbri et al., 2008), early detection of DEBs and associated emotional and behavior problems warrant immediate attention.

Therefore, this study aims to: (1) assess the prevalence of DEBs (including frequency of insulin misuse, IM) in a large Italian pediatric population; (2) explore potential demographic (gender, age, socioeconomic status [SES]), clinical quality of metabolic control, diabetes duration, type of insulin therapy, carb counting) and psychological differences (emotional and behavioral problems) among adolescents with and without DEBs, with and without IM.

In samples of youths with and without T1D, DEBs are described as more prevalent in female adolescents than male adolescents (Baechle et al., 2014; Neumark‐Sztainer et al., 2011), as well as occurring differently by teen age groups (Peducci et al., 2018); therefore, in this study, DEB prevalence was also stratified by gender and age.

## 2 METHODS

### 2.1 Sample selection and recruitment

The present study is part of the project DiabEaT1 (The *Disordered eating behaviors and behavioral and emotional problems in Italian adolescents with type 1 diabetes: A nationwide population‐based study*) a national, cross‐sectional study funded by the University of Campania “Luigi Vanvitelli”.

Of the *N* = 50 pediatric diabetes centers belonging to the Italian Society for Pediatric Endocrinology and Diabetology (ISPED), the *N* = 34 that were present at ISPED 2019 conference were asked to participate (not all centers were present at the conference). *N* = 4 declined mainly due to the lack of a psychologist on the diabetes center's team. All the centers that agreed to participate (*N* = 30) were asked to consecutively recruit all participants who met the following inclusion criteria: aged 11–19 years (both inclusive), diabetes onset at least 1 year before recruitment, being present with primary caregiver, and able to read and understand the questionnaire. Exclusion criteria included the presence of any comorbid developmental, cognitive, or psychiatric disorders (e.g., autism spectrum disorders, intellectual disabilities, mental retardation, psychosis) or medical conditions (e.g., celiac disease). Participants' clinical records were systematically examined to confirm that the inclusion/exclusion criteria were met.

All procedures performed in this study were approved by the Institutional Research Committee of the University of Campania “Luigi Vanvitelli” (Prot. N. 70 05/02/2020; 0012372/I, May 22, 2020) and by the Ethics Committees of all participating centers. Data collection started in January 2020, was suspended in March 2020 following the outbreak of COVID‐19, and re‐started on July 2020. This paper provides an overview of the mid‐term results that were obtained.

## 3 MEASURES

### 3.1 Sociodemographic and clinical data

A brief schedule was specifically designed and completed by clinicians (diabetologists or psychologists) to record demographic and clinical data, including age, gender, height, weight, quality of metabolic control (measured by current glycosylated hemoglobin, or HbA1c values), duration of illness, and other medical conditions. Possible missing data was obtained by reviewing medical charts.

#### 3.1.1 Weight status

BMI was used as a measure of the actual weight status and was calculated as weight/height^2^. BMI was transformed into Z‐scores using the Centers for Disease Control and Prevention's pediatric growth charts as a reference (Kuczmarski et al., 2002).

#### 3.1.2 Disordered eating behaviors

The Diabetes Eating Problems Survey‐R (DEPS‐r) is a diabetes‐specific measure of DEBs that includes food restriction/dysregulation, weight loss, insulin reduction/omission, and vomiting (Markowitz et al., 2010). The instrument is self‐administered and composed of 16 items (e.g., “I skip meals and/or snacks “; “I make myself vomit”) which are rated on a 6‐point Likert scale ranging from 0 (never) to 5 (always) indicating the frequency of the behavior. Higher scores indicate more DEBs. DEPS‐r scores of 20 or higher were considered to indicate a level of DEBs warranting further attention (Markowitz et al., 2010; Wisting et al., 2019). In line with the DEPS‐r factor structure (as identified in children and adolescents with T1D), prevalence of DEBs was also evaluated through maladaptive eating habits, preoccupation with thinness/weight, and the maintenance of high‐blood glucose to lose weight (Wisting et al., 2013b). The DEPS‐r has demonstrated satisfactory psychometric properties (Atik Altınok et al., 2017; Saßmann et al., 2015; Wisting et al., 2013a) and in Italian samples (Cherubini et al., 2018; Pinna et al., 2017), with good internal consistency (Cronbach's Alpha >0.8), test–retest reliability (inter‐class correlation coefficient >0.9), and convergent and criterion validity. A validated Italian version of the DEPS‐R was used in this study (Pinna et al., 2017). In the present study, DEPS‐r showed a good level of internal consistency (alpha Cronbach = .836).

In line with previous studies (Wisting et al., 2013a), insulin reduction and insulin omission were operationally defined using the answers given to DEPS‐r items 2 (“When I over‐eat, I do not take enough insulin to cover the food”) and 8 (“After I overeat, I skip my next insulin dose.”): answers indicating reducing or skipping at least “sometimes” over the last few months were classified as intentional IM.

#### 3.1.3 Behavioral and emotional problems

The Youth Self‐Report (YSR) is a 112‐item self‐report form in which adolescents rate their problems (Achenbach, 1991). Eight problem areas are addressed: somatic complaints, anxious/depressed behavior, social problems, thought problems, attention problems, delinquent behavior, aggressive behavior, and withdrawal. A Total Problems scale encompasses symptoms across these domains and can be further decomposed into two subscales: Internalizing Problems (i.e., withdrawn, anxiety/depressive, and somatic complaints) and Externalizing Problems (i.e., rule‐breaking, hyperactivity, and aggression). Participants rate the items (e.g., “I get in many fights;” “I worry a lot”) on a 3‐point Likert‐type scale. The responses to the YSR are summed to create scale scores. The YSR a gold standard in the assessment of clinical problems among youths; it is a well‐established measure with sound psychometric properties being reported in most countries (Achenbach & Rescorla, 2001). In the present study, the YSR subscales showed good internal consistency (alpha Cronbach = .670–.935).

#### 3.1.4 Socioeconomic status

The Barratt Simplified Measure of Social Status (BSMSS) (Barratt, 2006) is a measure of SES based on marital status, current employment status, and level of education. The measures' scores range from 8 to 66, with higher scores indicate higher socioeconomic levels.

### 3.2 Procedure

Patients were invited to participate in the study at the time of their routine clinic visit. Evaluations were individual and anonymous, in a quiet and comfortable room that was made available by the clinic. Those willing to participate were asked to sign a written informed consent form. The tests were administered by clinicians; adolescents were asked to fill out the DEPS‐r and the YSR, while parents were asked to fill out the BSMSS.

### 3.3 Statistical analysis

Cronbach's alpha (*α*) was computed to assess the homogeneity of the scales.

The prevalence of DEBs (i.e., DEPS‐r score ≥ 20) and the frequency of IM are presented as percentages. Group differences were assessed using the *x* ^2^ test for categorical variables and the *t*‐test for continuous variables. A one‐way ANOVA was used to examine the differences in demographic/clinical data and DEPS‐r scores between different age groups (three categories: 11–13 years, 14–16 years, and ≥ 17 years); post hoc comparisons were conducted using the Tukey test. The significance level was set at 0.05. To account for multiple comparisons and to minimize type 1 error, Bonferroni's correction was calculated by dividing *α* = .05 by the total number of tests carried out for each set of comparisons (boys vs. girls; early vs. middle vs. late adolescents; DEBs vs. no DEBs; IM vs. no IM). The corrected *α* was presented in the legend of each table.

For assessment of effect size, Cramer's *V* was calculated for the *x* ^2^ test (0.10 = small, 0.30 = medium, 0.50 = large), Cohen's *d* for the *t*‐test (>0.2 small, >0.5 medium, >0.8 large), and Partial eta‐squared (*η* ^*2*^) for the ANOVA (0.01 = small, 0.06 = moderate, >0.14 large) (Cohen, 1988).

To evaluate the relationship between DEBs and all measured demographic, clinical, and psychological variables, a linear stepwise regression analysis was conducted.

All analyses were conducted with raw scores. The statistical analysis was performed using Statistical Package for the Social Sciences (SPSS) version 21.0 for Macintosh.

## 4 RESULTS

### 4.1 Sample characteristics

Of 724 adolescents who were invited to participate, *n* = 4 declined (99.45% response rate) because of lack of time (*n* = 3) or interest in the study (*n* = 1). In a secondary analysis, *n* = 30 evaluated individuals were excluded because they did not meet the inclusion criteria (*n* = 20) or they returned incomplete assessments (*n* = 10). In total, 690 adolescents with T1D (353 boys) completed the study. N = 41 participants were evaluated before COVID‐19 outbreak.

In a comparison of boys and girls, no significant differences were found in terms of age, illness duration, SES, HbA1c values, type of therapy multiple daily injection/insulin pump/injection port, or use of carb counting (all *p* > .05 or not significant following Bonferroni correction); zBMI scores (*p* < .0001, *d* = .29) was higher in girls (Table 1).

**TABLE 1:** Demographic characteristics, clinical characteristics and DEPS‐r data of the whole sample, grouped by gender and age

	Whole sample *N* = 690	Boys *n* = 353	Girls *n* = 337	Boys vs. girls		Early vs. middle vs. late	
	*M(SD)*	*M(SD)*	*M(SD)*	*Test*	*p, effect size*	*Test*	*p, effect size*
Age (years, month) range	14.97 (1.81)(11–19.07)	14.98 (1.79)	14.95(1.81)	*t*(688) = .230	.818	—	—
SES	29.8 (10.98) (8.0–63.5)	30.09(10.98)	29.48(10.99)	*t*(688) = .723	.470	*F*(2,673) = 1.705	.183
HbA1c (%)	7.79 (1.25)	7.69(1.19)	7.9(1.31)	*t*(688) = −2.242	.025, *d* = −.17	*F*(2,690) = 1.663	.190
Diabetes duration (years)	6.79 (3.99)	6.88(4.07)	6.71(3.91)	*t*(688) = .566	.571	*F*(1,690) = 26.182	<.0001^a^, *η* ^*2*^ = .071
zBMI	.49 (1.01)	.35(1.15)	.64(.81)	*t*(688) = −3.956	.0001^b^, *d* = .29	*F*(2,687) = 3.815	.023, *η* ^*2*^ = .011
MDI (*n*, %)	406 (59)	219(62)	187(55.)	*x* ^2^ = 2.811	.245	*x* ^2^ = .5.355	.253
CSII (*n*, %)	275 (40)	131(37.1)	144(43)	*x* ^2^ = 2.811	.245	*x* ^2^ = .5.355	.253
Injection port (*n*, %)^c^	7 (1)	3(.8)	4(1.2)	*x* ^2^ = 2.811	.245	*x* ^2^ = .5.355	.253
Carb counting (yes) (*n*, %)^e^	384 (55.6)	198(56.1)	186(55.2)	*x* ^2^ = .102	.750	*x* ^2^ = .3.081	.214
DEPS‐r							
Total score	15.51(11.03)	13.26(9.45)	17.87(12.06)	*t*(688) = −5.596	<.0001^b^, *d* = .42	*F*(2,690) = .040	.961
Score ≥ 20 (*n*, %)	194 (28.1)	76 (21.5)	118(35)	*x* ^2^ = .15.512	<.0001^b^, *V* = .150	*x* ^2^ = .296,	.863
Insulin misuse (*n*, %)^d^	269(38.9)	141(39.9)	128(37.9)	*x* ^2^ = .232	.630	*x* ^2^ = 2.796	.247
Maladaptive eating habits	10.69(7.38)	9.89(6.8)	11.5(7.87)	*t*(682) = −2.847	.005, *d* = .23	*F*(2,683) = .044	.957
Preoccupation with thinness and weight	4.55(4.739	3.17(3.97)	5.99(5.02)	*t*(687) = −8.149	<.0001^b^, *d* = .62	*F*(2,688) = .173	.841
Maintaining high‐blood glucose to lose weight	.32(1.03)	.23(.81)	.41(1.21)	*t*(687) = −2.131	.034, *d* = .17	*F*(2,688) = .683	.505

*Note*: Data are presented as mean values and SD, unless otherwise stated.

Abbreviations: CSII, continuous subcutaneous insulin infusion; MDI, multiple day injection.

^a^ Significant after Bonferroni's correction (early vs. middle vs. late, corrected alpha for accepting statistical significance: .05/14 tests = .0036).

^b^ Significant after Bonferroni's correction (boys vs. girls, corrected alpha for accepting statistical significance: .05/15 tests = .0033).

^c^ The 2 (.3%) missing.

^d^ Answers indicating skipping or reducing at least “sometimes” were classified as insulin misuse.

^e^ Answers indicating carb counting at least once per day.

In a comparison of age groups, no significant differences were found in terms of SES, HbA1c values, zBMI, type of insulin therapy, or frequency of use of carb counting (all *p* > .05 or not significant following Bonferroni correction). Unsurprisingly, illness duration was longer in older participants (*p* < .001, *η* ^*2*^ = .071) (Table 1).

### 4.2 DEB prevalence

For the DEPS‐r scores, 28.1% of adolescents of T1D (21% boys, 35% girls) had values of 20 or more, indicating presence of DEBs. Girls had significantly higher DEPS‐r total scores (*p* < .0001, *d* = .42), primarily manifesting as preoccupation with thinness and weight (*p* < .0001, *d* = .62), and more frequent DEPS‐r scores of ≥20 (*p* = <.0001, *V* = .15) than boys; no gender differences were observed in IM (*p* > .05).

Across the whole sample, no age differences were observed in mean DEPS‐r total scores, and frequency of DEPS‐r scores ≥20 and of IM (all *p* > .05) (Table 1).

The analyses of each age group's results generally mirrored those observed in the whole sample—except for HbA1c values, which were found to be higher in older girls than boys (*p* < .0001, *d* = .61), and DEPS‐r scores/frequency of DEPS‐r scores ≥20, which were found to not be higher in girls than boys in the early adolescent group (both *p* > .05) (Table S1).

### 4.3 Adolescents with DEBs versus adolescents without DEBs, adolescents with IM versus adolescents without IM

Participants with DEBs had higher HbA1c (*p* < .0001, *d* = .54) and zBMI values (*p* < .0001, *d* = .52) in both genders (Table 2) and in all age groups—except for zBMI/HbA1c values in early adolescents, which did not differ between adolescents with and without DEBs (both *p* = .007, not significant following Bonferroni correction) (Table S2).

**TABLE 2:** Socio‐demographic characteristics, clinical data, and YSR scores in adolescents with T1D with and without DEBs

Total sample					Boys				Girls			
	DEBs *n* = 194	No DEBs *n* = 496	DEBs/no DEBs		DEBs *n* = 76	No DEBs *n* = 277	DEBs/no DEBs		DEBs *n* = 118	No DEBs *n* = 219	DEBs/no DEBs	
	*M(SD)*	*M(SD)*	*test*	*p*, *effect size*	*M(SD)*	*M(SD)*	*test*	*p*, *effect size*	*M(SD)*	*M(SD)*	*test*	*p*, *effect size*
Age: year, month	15.02(1.87)	14.94(1.77)	*t*(688) = .500	.617	14.89 (1.82)	15.01(1.79)	*t*(351) = −.528	.598	15.02(1.9)	14.87(1.77)	*t*(335) = 1.179	.239
SES	28.8(11.18)	30.19(10.88)	*t*(688) = −1.478	.140	28.14(9.12)	30.62(11.39)	*t*(351) = −1.963	.052	29.22(12.34)	29.63(10.19)	*t*(335) = −.302	.763
Hb1Ac (%)	8.29(1.38)	7.6(1.14)	*t*(688) = 6.168	<.0001^a^, *d* = .54	8.18(1.26)	7.55(1.13)	*t*(351) = 4.181	<.0001^a^, *d* = .53	8.36(1.46)	7.65(1.15)	*t*(335) = 4.517	<.0001^a^, *d* = .54
Duration of illness: year, month	6.78(3.93)	6.8(4.02)	*t*(688) = −.066	.947	6.99(4.1)	6.86(4.07)	*t*(351) = .268	.798	6.65(3.83)	6.75(3.95)	*t*(335) = −.221	.825
z‐BMI	.93(1.14)	.32(.9)	*t*(688) = 6.703	<.0001^a^, *d* = .52	.99(1.51)	.17(.95)	*t*(351) = 5.830	<.0001^a^, *d* = .65	.89(.82)	.52(.78)	*t*(335) = 4.129	<.0001^a^, *d* = .46
MDI/CSII/iport (*n*)	124/67/2	282/208/5	*x* ^2^ = 3.102	.212	54/22/0	165/109/3	*x* ^2^ = 3.830	.147	70/45/2	117/99/2	*x* ^2^ = 1.773	.412
Carb counting (yes) (*n*)^b^	88	296	*x* ^2^ = 11.516	.001, *V* = .130	33	165	*x* ^2^ = 5.638	.018, *V* = .127	55	131	*x* ^2^ = 5.859	.015, *V* = .132
YSR												
Withdrawn	5.31(3.24)	3.4(2.73)	*t*(688) = 7.282	<.0001^a^, *d* = .64	4.51(2.93)	3.22(2.64)	*t*(351) = 3.699	<.0001^a^, *d* = .46	5.83(3.34)	3.63(2.83)	*t*(335) = 6.083	<.0001^a^, *d* = .71
Somatic complaints	4.56(3.22)	2.68(2.42)	*t*(688) = 7.361	<.0001^a^, *d* = .66	3.59(2.73)	2.26(2.06)	*t*(351) = 3.940	<.0001^a^, *d* = .55	5.18(3.36)	3.20(2.71)	*t*(335) = 5.495	<.0001^a^, *d* = .65
Anxious/depressed	8.47(5.22)	5.01(3.55)	*t*(688) = 8.499	<.0001^a^, *d* = .77	6.11(4.22)	4.21(3.14)	*t*(351) = 3.642	<.0001^a^, *d* = .51	10.00(5.25)	6.02(3.78)	*t*(335) = 7.278	<.0001^a^, *d* = .87
Social problems	4.29(3.3)	2.5(2.34)	*t*(688) = 6.887	<.0001^a^, *d* = .62	3.51(2.59)	2.39(2.22)	*t*(351) = 3.747	<.0001^a^, *d* = .46	4.79(3.61)	2.64(2.47)	*t*(335) = 5.771	<.0001^a^, *d* = .70
Thought problems	4.45(4.24)	2.58(2.76)	*t*(688) = 5.679	<.0001^a^, *d* = .52	3.33(3.04)	2.48(2.58)	*t*(351) = 2.223	.028, d = .30	5.17(4.73)	2.71(2.99)	*t*(335) = 5.126	<.0001^a^, *d* = .62
Attention problems	7.53(3.3)	4.97(3.05)	*t*(688) = 9.662	<.0001^a^, *d* = .80	7.16(2.83)	5.00(3.13)	*t*(351) = 5.428	<.0001^a^, d = .72	7.76(3.56)	4.93(2.95)	*t*(335) = 7.373	<.0001^a^, *d* = .86
Rule‐breaking behavior	3.71(2.93)	2.47(2.23)	*t*(688) = 5.317	<.0001^a^, *d* = .48	4.13(2.88)	2.74(2.34)	*t*(351) = 3.875	<.0001^a^, *d* = .53	3.44(2.93)	2.14(2.05)	*t*(335) = 4.194	<.0001^a^, *d* = .51

	Total sample				Boys				Girls			
	DEBs *n* = 194	No DEBs *n* = 496	DEBs/no DEBs		DEBs *n* = 76	No DEBs *n* = 277	DEBs/no DEBs		DEBs *n* = 118	No DEBs *n* = 219	DEBs/no DEBs	DEBs *n* = 118
	*M(SD)*	*M(SD)*	*test*	*p, effect size*	*M(SD)*	*M(SD)*	*test*	*p, effect size*	*M(SD)*	*M(SD)*	*test*	*M(SD)*
Aggressive behavior	9.81(4.91)	6.87(3.92)	*t*(688) = 7.482	<.0001^a^, *d* = .66	9.30(4.57)	6.86(3.98)	t(351) = 4.586	<.0001^a^, *d* = .57	10.14(5.1)	6.88(3.86)	*t*(335) = 6.080	<.0001^a^, *d* = .72
Internalizing	18.35(10.21)	11.08(7.11)	*t*(688) = 9.082	<.0001^a^, *d* = .83	14.21(8.5)	6.69(6.39)	t(351) = 4.309	<.0001^a^, *d* = 1.00	21.01(10.36)	12.84(7.58)	*t*(335) = 7.541	<.0001^a^, *d* = .90
Externalizing	13.53(7.02)	9.34(5.05)	*t*(688) = 7.457	<.0001^a^, *d* = .68	13.43(6.77)	9.60(5.7)	t(351) = 4.518	<.0001^a^, *d* = .61	13.58(7.2)	9.01(5.25)	*t*(335) = 6.080	<.0001^a^, *d* = .72
Total problems	73.95(24.55)	54.45(17.96)	*t*(688) = 10.059	<.0001^a^, *d* = .91	66.33(20.63)	52.86(17.86)	t(351) = 5.627	<.0001^a^, *d* = .70	78.86(25.68)	56.47(17.92)	*t*(335) = 8.429	<.0001^a^, *d* = 1.01

*Note*: Data are presented as mean values and SD, unless otherwise stated; MDI = multiple day injection; CSII = continuous subcutaneous insulin infusion; iport = Injection port.

^a^ Significant after Bonferroni's correction (corrected alpha for accepting statistical significance: .05/18 tests = .0027).

^b^ Answers indicating carb counting at least once per day.

In the whole sample, no differences were found between participants with and without DEBs regarding age, duration of illness, SES, and insulin therapy, whether between boys and girls (Table 2) or across age groups (all *p* > .05 or not significant following Bonferroni correction) (Table S2). Additionally, the frequency of using a carb counting system was significantly lower in groups with DEBs (*p* = .001, *V* = .130) (Table 2).

Participants with DEBs showed higher emotional and behavioral problems for all dimensions considered (all *p* < .0001, *d* range = .51–1.1); the same occurred in the analysis of boys and girls within the sample (all *p* < .0001) (except for boy Thought problems, *p* = .028, which was not significant following Bonferroni's correction) (Table 2), and in the analysis of early, middle, and late adolescents (all *p* < .05, *d* range: .37–1.43) (except for Thought Problems in early adolescents, Social problems, Rule‐breaking behaviors, Aggressive behavior, Externalizing in late adolescents; *p* > .05 or not significant following Bonferroni's correction, Table S2).

The mean DEPS‐r scores, HbA1c values, and emotional and behavioral problems subscale scores of adolescents who misused insulin were found to be significantly higher than those of adolescents who did not misuse insulin (all *p* < .001, *d* range: .26–1.07). No differences were found with regard to age, SES, duration of illness, zBMI, type of insulin therapy, use of a carb counting system (all *p* > .05 or not significant following Bonferroni correction) (Table 3).

**TABLE 3:** Socio‐demographic, clinical data, and YSR scores in adolescents with T1D with and without insulin misuse behaviors

	Insulin misuse *n* = 269	No insulin misuse *n* = 421	Insulin misuse/no insulin misuse	
	*M(SD)*	*M(SD)*	*Test*	*p*, *effect size*
Age: year, month	14.89(1.87)	15.02(1.76)	*t*(687) = −.902	.367
SES	29.53(10.89)	30.01(11.03)	*t*(687) = −.557	.578
Hb1Ac (%)	7.99(1.3)	7.66(1.2)	*t*(687) = 3.366	.001^a^, *d* = .26
Duration of illness: year, month	6.78(3.94)	6.8(4.02)	*t*(687) = −.061	.952
z‐BMI	.52(1.17)	.48(.88)	*t*(687) = .428	.669
MDI/CSII/iport (*n*)	156/108/3	249/167/4	*x* ^2^ = .087	.958
Carb counting (yes) (*n*)^b^	134	249	*x* ^2^ = 5.337 (RS = 1.4)	.021, *V* = .088
Tot DEPS‐r	22.16(11.84)	11.28(8.03)	*t*(687) = 13.231	<.0001^a^, *d* = 1.07
YSR				
Withdrawn	4.64(3.1)	3.48(2.87)	*t*(687) = 5.002	<.0001^a^, *d* = .38
Somatic complaints	3.69(2.9)	2.9(2.69)	*t*(687) = 3.641	<.0001^a^, *d* = .28
Anxious/depressed	6.92(4.68)	5.38(4.07)	*t*(687) = 4.415	<.0001^a^, *d* = .35
Social problems	3.71(2.84)	2.55(2.61)	*t*(687) = 5.349	<.0001^a^, *d* = .42
Thought problems	3.65(3.47)	2.76(3.24)	*t*(687) = 3.394	.001^a^, *d* = .26
Attention problems	6.78(3.32)	5.00(3.14)	*t*(687) = 7.098	<.0001^a^, *d* = .55
Rule‐breaking behavior	3.43(2.6)	2.43(2.37)	*t*(687) = 5.182	<.0001^a^, *d* = .4
Aggressive behavior	8.69(4.45)	7.06(4.3)	*t*(687) = 4.800	<.0001^a^, *d* = .37
Internalizing	15.25(9.16)	11.77(8.18)	*t*(687) = 5.071	<.0001^a^, *d* = .41
Externalizing	12.13(6.32)	9.49(6.01)	*t*(687) = 5.496	<.0001^a^, *d* = .43
Total problems	66.41(22.5)	55.79(20.43)	*t*(687) = 6.396	<.0001^a^, *d* = .49

*Note*: Data are presented as mean values and SD, unless otherwise stated.

Abbreviations: CSII, continuous subcutaneous insulin infusion; iport, injection port; MDI, multiple day injection.

^a^ Significant after Bonferroni's correction (corrected alpha for accepting statistical significance: .05/19 tests = .0026).

^b^ Answers indicating carb counting at least once per day.

In an analysis of the data organized by age group, adolescents who misused insulin did not substantially differ from adolescents who did not misuse insulin in terms of their demographic and clinical characteristics (all *p* > .05 or not significant following Bonferroni correction), but they showed higher DEPS‐r scores and—especially those in the early and middle groups—more emotional and behavioral problems (Table S3).

In terms of the relationships between DEBs and all measured demographic, clinical, and psychological variables, in a stepwise regression analysis applied to the whole sample, YSR Total Problems, HbA1c, zBMI, and gender explained 39.2% of DEPS‐r variance. YSR Total Problems was the most influential variable in predicting DEBs (*β* = .455, *p* < .0001) (Table 4).

**TABLE 4:** Results of stepwise linear regression analysis examining the associations of DEPS‐r scores with demographic, clinical, and psychological variables across the whole sample

	Regression coefficient *β*	*p* value	*R* ^2^ change	Adjusted *R* ^2^
Predictors^a^				
YSR‐total problems	.455	<.0001	.277	.275
HbA1c	.272	<.0001	.088	.363
zBMI	.154	<.0001	.025	.388
Gender (f)^b^	.074	.017	.005	.392

*Note*: DEPS‐r score as dependent variable; gender, age, SES, duration of illness, zBMI, HbA1c, internalizing, externalizing, and total Problem as independent variables.

^a^ A variable was included in the model when its *p* value was <.05 and was excluded when it was >.01; only variables significantly contributing to the models are displayed.

^b^ Gender was treated as a continuous variable.

## 5 DISCUSSION

This study is the first to assess the prevalence of DEBs and IM in a large national Italian sample of adolescents with T1D. The prevalence of DEBs found here (about 28.1%) is comparable to previous reports on adolescents with T1D, from Italian (34.4%, Cherubini et al., 2018; 37.7%, Troncone, Cascella, Chianese, Zanfardino, Piscopo, et al., 2020; 36.5%, Troncone, Chianese, Zanfardino, Cascella, Confetto et al., 2020b) and non‐Italian samples (38%, Araia et al., 2017; 21.2%, Nip et al., 2019; 18.3%, Wisting et al., 2013a). Similarly, in line with cross‐sectional and longitudinal studies in adolescents with T1D (Araia et al., 2017, Troncone, Cascella, Chianese, Zanfardino, Piscopo, et al., 2020; Troncone, Chianese, Zanfardino, Cascella, Confetto et al., 2020a; Wisting et al., 2013a) and in the general population (Neumark‐Sztainer et al., 2011), girls—especially those in middle and late adolescence—showed a higher prevalence of DEBs (mainly as preoccupation with thinness/weight) than boys, as well as higher zBMI and poorer glycemic control (Minges et al., 2017).

As previously described, DEBs were found to be unrelated to the type of treatment (including when the sample was grouped by gender) and the use of carb counting was found to be less frequent in group with DEBs (Cherubini et al., 2018; Prinz et al., 2016).

In addition, early, middle, and late adolescents did not differ from each other in DEB prevalence and in most demographic and clinical features. Previous results on differences in DEB prevalence among age groups are contradictory, suggesting either fewer eating problems in early teens and increasing DEB prevalence with age (Nip et al., 2019; Wisting et al., 2013a) or no age differences (Scheuing et al., 2014). Such results indicate that this issue needs to be further explored.

In comparisons of adolescents with and without DEBs, adolescents with eating problems showed adverse clinical and psychological conditions, characterized by higher zBMI, poorer metabolic control, and higher emotional and behavioral problems. In a comparison of adolescents with and without DEBs grouped by gender or age, the results largely mirrored those found in the whole sample. Moreover, emotional/behavioral problems, HbA1c, zBMI, and gender were identified as main statistical predictors of DEBs.

With regard to clinical data, higher HbA1c/BMI values observed in adolescents with DEBs in this study are in line with the association between worse metabolic control/higher weight and disordered eating that is generally described in several reviews focusing on DEBs in adolescents with T1D (Conviser et al., 2018; Pinhas‐Hamiel et al., 2015; Wagner & Karwautz, 2020; Young et al., 2013). In particular, considering the BMI–DEBs association, it is reasonable to suppose a circular relationship, where higher weight may result in higher body dissatisfaction and greater desire to lose weight, which in turn may push adolescents (already struggling with developmental issues of self and body image concerns) to adopt unhealthy strategies (e.g., skipping meals, purging behaviors); suffering from DEBs may result in gaining weight (Neumark‐Sztainer et al., 2012) and seriously affect metabolic outcomes (Broadley et al., 2020; Winston, 2020).

In fact, poor glycemic control is described as one of the first signs of presence of DEBs (Toni et al., 2017).

In addition, adolescents with DEBs showed both higher internalizing and externalizing symptoms, which have been clearly indicated in previous studies with children and adolescents as playing a negative role in diabetes management, as favoring/predicting poor metabolic control, and as being associated with psychiatric and health morbidity (Bryden et al., 2001; Cohen et al., 2004; Luyckx et al., 2010; McDonnell et al., 2007; Northam et al., 2005). It is likely that the DEBs and psychological problems are mutually exacerbating (Broadley et al., 2020).

Finally, with regard to IM prevalence, our findings of 38.9% of participants admitting to IM are higher than the majority of prevalence estimates indicated in other studies with adolescents (Bächle et al., 2016; Cherubini et al., 2018; Pinhas‐Hamiel et al., 2015; Schober et al., 2011; Snyder et al., 2016; Wagner & Karwautz, 2020; Wisting et al., 2013a), although they are consistent with recent research indicating no significant gender differences in IM frequency (Araia et al., 2017; Snyder et al., 2016; Troncone, Chianese, Zanfardino, Cascella, Confetto et al., 2020b).

In line with previous evidence (Araia et al., 2017; Wisting et al., 2013a), in a comparison of adolescents with and without IM, the HbA1c values, DEPS‐r mean scores, and emotional/behavioral problems were found to be higher in the IM group.

However, any interpretation of our findings should take into account the definitions of insulin reduction/omission. How we defined IM, and the fact that the two DEPS‐R questions asking about insulin reduction/omission did not specify that such behavior is for the purpose of weight loss, could have potentially biased IM estimation. Specifically, the IM prevalence reported here could have been inflated by the broad inclusion criterion for defining IM that was employed in the present study, which risks pathologizing individuals reporting occasional IM. However, considering the wide variation in IM evaluation methods in existing studies (e.g., patient‐report IM frequency, answers given to DEPS‐r items, direct clinical evaluation) (Araia et al., 2017; Bächle et al., 2016; Cherubini et al., 2018; Snyder et al., 2016; Troncone, Chianese, Zanfardino, Cascella, Confetto et al., 2020a; Wisting et al., 2013a), all comparisons of the present results with previous research findings about IM should be viewed with caution.

The large population‐based sample, recruited from 30 pediatric centers across the country, is a strength of this study. In addition, both boys and girls were included, and DEBs were assessed with a diabetes‐specific measure, which ensured a direct evaluation of purging behaviors unique to T1D, such as insulin omission/reduction. Moreover, the evaluation of DEBs carried out in the present study—that is, according to DEPS‐r factors—provides information about scores on maladaptive eating habits, preoccupation with thinness/weight, and the maintaining of high‐blood glucose to lose weight, which can extend the existing literature on DEBs' characteristics in adolescents with T1D.

In this study, in addition to the possible contributions for those working with EDs and in the T1D field who seek a deeper understanding of co‐occurring DEBs–emotional/behavioral problems in youths with T1D, a number of limitations should also be acknowledged. The first limitation stems from the use of self‐report measures for DEBs and for emotional and behavioral problems. The limitations of the measurements should be considered, especially when interpreting gender differences in DEB occurrence, due to a recognized bias across eating disorder tools, which are frequently focused on eating disorder symptoms that are more salient among girls/women (Pursey et al., 2020).

The cross‐sectional nature of this study and the exclusion of participants with cognitive, developmental, and psychological problems should be considered as other limitations. The exclusion of youths with T1D with mental health problems—who are likely to also be more vulnerable to DEBs—may have introduced a possible sample selection bias that limited the generalizability of the study and probably affected the results (especially the YSR data).

Significantly, the COVID‐19 pandemic was occurring at the time of this study, thus, the present findings about emotional and behavior problems could somehow be influenced by the stress that was generally observed due to the outbreak. Therefore, it is important for future research to study eating behaviors, associated emotional and behavioral problems, and co‐occurring mental health disorders after the pandemic has ended. Given the young age of participants, it would also be interesting for a more complete analysis to examine DEBs and emotional/behavioral problems using a parent‐report assessment.

Lastly, this study carried out only a preliminary exploration of the relationships between DEBs and clinical and psychological factors. As the data collection for DiabEaT1 continues, an interesting next step would be to investigate the relationships between DEBs and demographic/clinical and psychological variables within a multivariate framework. Through structural equation modeling (SEM), the possible indirect and direct pathways between DEBs, demographic/clinical variables, and emotional/behavioral problems in adolescents can be examined.

In conclusion, given the relationship between DEBs and an increased risk of diabetes complications (i.e., retinopathy, neuropathy, and other microvascular complications) (Broadley et al., 2020; Winston, 2020), the worse clinical status and higher emotional/behavioral problems demonstrated by adolescents with DEBs, and the potential influence of adolescents' internalizing and externalizing problems on their diabetes management and control, the present results emphasize the importance of continuously monitoring the clinical and psychological condition of youth with T1D (Delamater et al., 2018). Health care providers should be aware of the increased risk of DEBs in adolescents with T1D and of the iatrogenic effect of some diabetes self‐care behaviors (such as focus on food intake and eating patterns, attention to weight, dietary regimen) which, with such an emotionally and cognitively demanding disease as T1D, may further increase that risk. Particular attention should be paid to BMI levels and glycemic control quality as possible indicators of DEB onset, as well as to the adolescent's general psychological condition. This appears particularly important considering the challenges and demanding tasks (e.g., increased involvement with peers, growing independence from parents) entailed in this critical developmental phase, which may further interfere with adherence to the diabetes regimen (Helgeson et al., 2009). Similarly, health care providers should also be aware of the potential for such problems in both genders, as well as of the underlying reasons and scopes of DEB symptoms. For example, possible IM episodes should not necessarily be viewed as a sign of DEB onset: to be properly managed, these episodes need to be understood within the context in which they appear (e.g., potential significant/stressful emotional events occurring within the relational/familial context), and other possible reasons for why individuals reduce or omit insulin (e.g., fear of hypoglycemia, interference with activities of daily living, injection pain/embarrassment, etc.) need to be ruled out.

Thus, during routine psychological assessment, it is important to combine screening measures with diagnostic interviews, conducted by experienced clinicians, to appropriately target the interview questions in order to ensure that DEBs are accurately screened and located within the more general comprehension of youth psychological functioning. To detect emerging difficulties early, both in eating behaviors and in general psychological functioning; to prevent T1D management's iatrogenic effects; and to facilitate treatment recommendation, it would be useful for clinicians engaged with diabetes care: (1) to work in a multidisciplinary team in which psychologists are involved in T1D patients' care/management; and (2) to continually expand their knowledge about eating disorders and co‐occurring psychological problems, as reported in the scientific literature.

## AUTHOR CONTRIBUTIONS

**Alda Troncone:** Conceptualization; formal analysis; funding acquisition; methodology; writing – original draft; writing – review and editing. **Gaetana Affuso:** Conceptualization; formal analysis; methodology; writing – review and editing. **Crescenzo Cascella:** Data curation; formal analysis; investigation; software; writing – review and editing. **Antonietta Chianese:** Data curation; formal analysis; investigation; software; writing – review and editing. **Angela Zanfardino:** Conceptualization; data curation; investigation; writing – review and editing. **Barbara Pizzini:** Data curation; formal analysis; investigation; software; writing – review and editing. **Dario Iafusco:** Conceptualization; funding acquisition; methodology; supervision; writing – review and editing.

## FUNDING INFORMATION

The research leading to these results received funding from the project, DiabEaT1, which received funding from University of Campania “Luigi Vanvitelli” through the programme V: ALERE 2019, funded with D.R. 906 del 4/10/2019, prot. n. 157264, October 17, 2019.

## CONFLICT OF INTEREST

The authors have no conflict to declare.

## Supporting information


**Table S1** Demographic characteristics, clinical characteristics, and DEPS‐r data of the sample, grouped by gender and age

## Data Availability

Data is available upon reasonable request.

## Collaborators of the Diabetes Study Group of the Italian Society for Pediatric Endocrinology and Diabetology (ISPED)

### 1

The following people were actively involved in the collection of data and in manuscript revision and have to be considered as coauthors of the paper: Riccardo Lera MD, Giulia Patrizia Bracciolini MD, Caterina Grosso MD, Enrica Bertelli MD (Alessandria); Valentino Cherubini MD (Ancona); Elvira Piccinno MD, Maurizio Delvecchio MD, Federica Ortolani MD, Marcella Vendemiale PsyD, Alessandra Rutigliano PsyD (Bari); Clara Zecchino MD (Bari); Stefano Zucchini MD, Giulio Maltoni MD, Dorella Scarponi PsyD, Lucia Fraternale PsyD (Bologna); Francesco Gallo MD, Maria Susanna Coccioli MD, Vito Brugnola PsyD (Brindisi); Carlo Ripoli MD, Maria Rossella Ricciardi MD, Sabrina Maria Galassi MD (Cagliari); Filomena Pascarella MD, Arcangelo Perrotta MD, Anna Golino PsyD (Caserta); Filomena Andreina Stamati MD (Castrovillari); Donatella Lo Presti MD, Manuela Caruso Nicoletti MD, Annalisa Saggio PsyD (Catania); Felice Citriniti MD (Catanzaro); Domenico Sperlì MD, Rosaria De Marco MD, Maria Daniela Borselli PsyD (Cosenza); Nicola Lazzaro MD (Crotone); Valeria De Donno MD, Cristina Giordana PsyD (Cuneo); Sonia Toni MD, Verena Balbo PsyD (Florence); Giuseppe d'Annunzio MD, Nicola Minuto MD, Marta Bassi MD, Alice Parodi PsyD (Genoa); Mimma Caloiero MD, Monica Aloe MD, Maria Corsini PsyD (Lamezia Terme); Rosanna Lia MD (Locri); Fortunato Lombardo MD, Giuseppina Salzano MD, Stefano Passanisi MD, Daniela Pecoraro PsyD (Messina); Riccardo Bonfanti MD, Clara Pozzi PsyD (Milan); Stefano Curto MD, Alessia Piscopo MD, Emanuele Miraglia del Giudice MD, Veronica Testa NDi, Assunta Serena Rollato NDi, Alessandro Pennarella MD (Naples); Ivana Rabbone MD, Ciro Pignatiello PsyD, Silvia Savastio MD, Valentina Antoniotti RDN (Novara); Fiorella De Berardinis MD, Giacomo Santoro MD (Paola Cetraro); Brunella Iovane MD, Silvia Dioni PsyD (Parma); Maria Carmela Lia MD, Rita Tutino PsyD (Reggio Calabria); Stefano Cianfarani MD, Riccardo Schiaffini MD, Ippolita Patrizia Patera MD, Maria Cristina Matteoli MD, Chiara Carducci PsyD (Rome); Irene Rutigliano MD, Grazia D'onofrio PsyD (San Giovanni Rotondo, FG); Luisa de Santis MD, Michela Trada MD, Davide Tinti MD, Cinzia Montarulo PsyD (Turin); Giuliana Cardinale MD, Sofia De Leo PsyD (Casarano, Gallipoli); Claudia Arnaldi MD; Barbaro Longo PsyD (Viterbo).
