# Supplementary material for: Prevalence of disordered eating behaviors in adolescents with type 1 diabetes: Results of multicenter Italian nationwide study
Source: Int J Eat Disord. 2022 Jun 25;55(8):1108–19. doi: 10.1002/eat.23764 (PMC9544556; doi:10.1002/eat.23764)
Supplement: Supplementary file 1 — Table S1 Demographic characteristics, clinical characteristics, and DEPS‐r data of the sample, grouped by gender and age [file EAT-55-1108-s001.docx]

**Table S1**

*Demographic characteristics, clinical characteristics and DEPS-r data of the sample, grouped by gender and age*

|  | Whole sample *N*=690 | Boys *n*=353 | Girls *n*=337 | Boys vs. Girls | |
| --- | --- | --- | --- | --- | --- |
|  | *M(SD)* | *M(SD)* | *M(SD)* | *test* | *p, effect size* |
|  |  |  |  |  |  |
| Early: 11-13 years | *n*=177 | *n*=90 | *n*=87 |  |  |
| Age (years, month) range | 12.72(.65) | 12.7(.69) | 12.75(.62) | *t*(175)=-.523 | .601 |
| SES | 29.45(10.28) | 30.01(10.35) | 28.87(10.23) | *t*(175)=.732 | .465 |
| HbA1c (%) | 7.87(1.2) | 7.96(1.21) | 7.78(1.21) | *t*(175)=-1.730 | .085 |
| Diabetes duration (years) | 5.58 (3.27) | 5.54(3.16) | 5.6(3.4) | *t*(175)=-.175 | .862 |
| zBMI | .46(.92) | .35(.99) | .59(.83) | *t*(175)=-1.724 | .085 |
| MDI (*n*, %) | 112(63.6) | 61(67.8) | 51(59.3) | X^2^=1.383 | .501 |
| CSII (*n*, %) | 62(35.2) | 28(31.1) | 34 (39.5) | X^2^=1.383 | .501 |
| Injection port (*n*, %)^a^ | 2(1.1) | 1(1.1) | 1(1.2) | X^2^=1.383 | .501 |
| Carb counting (yes) (*n*, %)^b^ | 106 (59.9) | 56(63.6) | 50(57.5) | X^2^=.696 | .404 |
| DEPS-r |  |  |  |  |  |
| Total score | 15.33(10.75) | 14.49(10.12) | 16.20(11.35) | *t*(175)=-1.056 | .292 |
| Score ≥ 20 (*n*, %) | 52(29.4) | 23(25.6) | 29 (33.3) | X^2^=1.290 | .256 |
| Insulin misuse (*n*, %) | 78(44.1) | 40(44.4) | 38(43.7) | X^2^=.011 | .918 |
|  |  |  |  |  |  |
| Middle: 14-16 years | *n*=356 | *n*=185 | *n*=171 |  |  |
| Age (years, month) range | 14.98(.81) | 15.05(.79) | 14.91(.83) | *t*(354)=1.625 | .105 |
| SES | 30.49(11.42) | 30.86(11.77) | 30.08(11.04) | *t*(354)=.637 | .525 |
| HbA1c (%) | 7.82(1.25) | 7.73(1.19) | 7.91(1.31) | *t*(354)=-1.315 | .189 |
| Diabetes duration (years) | 6.62(3.77) | 6.87(3.89) | 6.34(3.63) | *t*(354)=1.327 | .185 |
| zBMI | .59(1.03) | .46(1.22) | .72(.75) | *t*(354)=-2.479 | .014, *d*=.26 |
| MDI (*n*, %) | 208(58.6) | 109(58.9) | 99(58.2) | X^2^=.301 | .860 |
| CSII (*n*, %) | 142(40) | 74(40) | 68(40) | X^2^=.301 | .860 |
| Injection port (*n*, %)^a^ | 5(1.4) | 2(1.1) | 3(1.8) | X^2^=.301 | .860 |
| Carb counting (yes) (*n*, %)^b^ | 199 (55.9) | 106(57.3) | 93(55) | X^2^=.185 | .668 |
| DEPS-r |  |  |  |  |  |
| Total score | 15.61(10.99) | 13.35(9.28) | 18.06(12.13) | *t*(354)=-4.086 | <.0001^c^, *d*=.43 |
| Score ≥ 20 (*n*, %) | 97(27.2) | 40(21.6) | 57(33.3) | X^2^=6.149 | .013, *V*=.131 |
| Insulin misuse (*n*, %) | 131(36.8) | 71(38.4) | 60(35.1) | X^2^=.334 | .564 |
|  |  |  |  |  |  |
| Late: 17-19 years | *n*=157 | *n*=78 | *n*=79 |  |  |
| Age (years, month) range | 17.46(.6) | 17.46(.57) | 17.47(.63) | *t*(155)=-.057 | .955 |
| SES | 28.57(10.66) | 28.31(9.51) | 28.83(11.78) | *t*(155)=-.294 | .770 |
| HbA1c (%) | 7.64(1.29) | 7.26(1.02) | 8.01(1.42) | *t*(155)=-3.826 | p<.0001^c^, *d*=.61 |
| Diabetes duration (years) | 8.59(4.56) | 8.46(4.84) | 8.71(4.29) | *t*(155)=-.337 | .736 |
| zBMI | .32(1.01) | .09(1.08) | .55(.89) | *t*(155)=-2.899 | .004^c^, *d*=.52 |
| MDI (n, %) | 86(54.8) | 49(62.8) | 37(46.8) | X^2^=4.048 (all RS <2) | .044 (all RS <2) |
| CSII (*n*, %) | 71(45.2) | 29(37.2) | 42(53.2) | X^2^=4.048 (all RS <2) | .044 (all RS <2) |
| Injection port (*n*, %)^a^ | 0 | 0 | 0 | X^2^=4.048 (all RS <2) | .044 (all RS <2) |
| Carb counting (yes) (*n*, %)^b^ | 79(50.3) | 36(47.4) | 43(54.4) | X^2^=.773 | .379 |
| DEPS-r |  |  |  |  |  |
| Total score | 15.49(11.52) | 11.64(8.89) | 19.29(12.57) | *t*(155)=-4.407 | <.0001^c^, *d*=.7 |
| Score ≥ 20 (*n*, %) | 45(28.7) | 13(16.7) | 32(40.5) | X^2^=10.909 | .001^c^, *V*=.264 |
| Insulin misuse (*n*, %)^d^ | 60(38.2) | 30(38.5) | 30(37.9) | X^2^=.004 | .950 |

*Note*: Data are presented as mean values and standard deviation, unless otherwise stated; MDI=multiple day injection; CSII=continuous subcutaneous insulin infusion

^a^ 2 (.3%) missing.

^b^ Answers indicating carb counting at least once per day.

^c^ Significant after Bonferroni’s correction (corrected alpha for accepting statistical significance: .05/12 tests = .004).

^d^ Answers indicating skipping or reducing at least “sometimes” were classified as insulin misuse.

**Table S2**

*Socio-demographic characteristics, clinical data, and YSR scores in adolescents with T1D with and without DEBs grouped by gender and age*

| Total sample | | | | | Boys | | | | Girls | | | |
| --- | --- | --- | --- | --- | --- | --- | --- | --- | --- | --- | --- | --- |
|  | DEBs  *n*=194 | No DEBs  *n*=496 | DEBs/no DEBs |  | DEBs  *n*=76 | No DEBs  *n*=277 | DEBs/no DEBs |  | DEBs  *n*=118 | No DEBs  *n*=219 | DEBs/no DEBs | |
|  | *M(SD)* | *M(SD)* | *test* | *p, effect size* | *M(SD)* | *M(SD)* | *test* | *p, effect size* | *M(SD)* | *M(SD)* | *test* | *p, effect size* |
|  |  |  |  |  |  |  |  |  |  |  |  |  |
| Early: 11-13 years | *n*=52 | *n*=125 |  |  | *n*=23 | *n*=67 |  |  | *n*=29 | *n*=58 |  |  |
| Age: year, month | 12.63(.75) | 12.76(.61) | *t*(175)=-1.110 | .270 | 12.65(7.1) | 12.71 | *t*(88)=-.330 | .742 | 12.61(.78) | 12.81(.51) | *t*(85)=-1.292 | .204 |
| SES | 27.31(9.54) | 30.35(10.48) | *t*(175)=-1.801 | .074 | 27.00(8.31) | 31.05(10.84) | *t*(88)=-1.632 | .106 | 27.55(10.55) | 29.53(10.08) | *t*(85)=-.849 | .398 |
| Hb1Ac (%) | 8.26(1.39) | 7.72(1.09) | *t*(175)=2.736 | .007, *d*=.43 | 8.39(1.55) | 7.82(1.04) | *t*(88)=1.981 | .05, *d*=.43 | 8.15(1.28) | 7.6(1.14) | *t*(85)=2.020 | .046, *d*=.45 |
| Duration of illness: year, month | 5.71(3.42) | 5.53(3.22) | t(175)=.330 | .742 | 6.48(3.88) | 5.21(2.83) | *t*(88)=1.445 | .159 | 5.09(2.92) | 5.89(3.61) | *t*(85)=-1.116 | .269 |
| z-BMI | .76(.84) | .34(.93) | *t*(175)=2.749 | .007, *d*=.47 | .7(1.01) | 2.23(.96) | *t*(88)=1.996 | .049, *d*=1.55 | .8(.7) | .48(.88) | *t*(85)=1.704 | .092 |
| MDI/CSII/iport (*n*) | 31/21/0 | 81/41/2 | X^2^=1.584 | .453 | 15/8/0 | 46/20/1 | X^2^=.507 | .776 | 16/13/0 | 35/21/1^a^ | *t*(85)=.945 | .624 |
| Carb counting (yes) (*n*)^b^ | 26 | 80 | X^2^=2.153 | .142 | 13 | 43 | X^2^=.036 | .850 | 13 | 37 | *t*(85)=.2845 | .092 |
|  |  |  |  |  |  |  |  |  |  |  |  |  |
| YSR |  |  |  |  |  |  |  |  |  |  |  |  |
| Withdrawn | 5.42(3.24) | 3.02(2.73) | *t*(175)=5.056 | <.0001^c^, *d*=.80 | 6.04(2.96) | 2.96(2.74) | *t*(88)=4.564 | <.0001^c^, *d*=1.08 | 4.93(3.41) | 3.09(2.73) | *t*(85)=2.730 | .008, *d*=.59 |
| Somatic complaints | 4.98(3.25) | 2.54(2.25) | *t*(175)=4.952 | <.0001^c^, *d*=.87 | 5.09(3.36) | 2.25(1.84) | *t*(88)=3.855 | .001^c^, *d*=1.05 | 4.9(3.22) | 2.86(2.62) | *t*(85)=3.154 | .002^c^, *d*=.69 |
| Anxious/depressed | 8.58(4.9) | 4.85(3.87) | *t*(175)=4.889 | <.0001^c^, *d*=.84 | 8.04(4.43) | 4.22(3.52) | *t*(88)=4.196 | <.0001^c^, *d*=.95 | 9.00(5.28) | 5.57(4.16) | *t*(85)=3.308 | .001^c^, *d*=.72 |
| Social problems | 4.65(3.34) | 2.68(2.44) | *t*(175)=3.853 | <.0001^c^, *d*=.67 | 4.57(3.01) | 2.93(2.62) | *t*(88)=2.492 | .015, *d*=.58 | 4.72(3.63) | 2.4(2.2) | *t*(85)=3.171 | .003, *d*=.77 |
| Thought problems | 4.54(4.81) | 2.57(2.5) | *t*(175)=2.803 | .007, *d*=.51 | 4.09(3.68) | 2.40(2.34) | *t*(88)=2.057 | .049, *d*=.54 | 4.90(5.58) | 2.76(2.66) | *t*(85)=2.425 | .017, *d*=.49 |
| Attention problems | 8.25(3.2) | 4.82(3.18) | *t*(175)=6.501 | <.0001^c^, *d*=1.07 | 7.35(2.42) | 5.16(3.51) | *t*(88)=2.758 | .007, *d*=.73 | 8.97(3.58) | 4.43(2.73) | *t*(85)=6.560 | <.0001^c^, *d*=1.43 |
|  | Total sample | | | | Boys | | | | Girls | | | |
|  | DEBs  *n*=194 | No DEBs  *n*=496 | DEBs/no DEBs |  | DEBs  *n*=76 | No DEBs  *n*=277 | DEBs/no DEBs |  | DEBs  *n*=118 | No DEBs  *n*=219 | DEBs/no DEBs | DEBs  *n*=118 |
|  | *M(SD)* | *M(SD)* | *test* | *p, effect size* | *M(SD)* | *M(SD)* | *test* | *p, effect size* | *M(SD)* | *M(SD)* | *test* | *M(SD)* |
| Rule-breaking behavior | 3.23(2.36) | 2.00(1.77) | *t*(175)=3.378 | .001^c^, *d*=.59 | 3.52(2.81) | 2.27(1.91) | *t*(88)=2.388 | .019, *d*=.52 | 3.00(1.96) | 1.69(1.56) | *t*(85)=3.384 | .001^c^, *d*=.74 |
| Aggressive behavior | 9.98(3.82) | 6.54(3.86) | *t*(175)=5.412 | <.0001^c^, *d*=.89 | 9.61(4.03) | 6.64(3.84) | *t*(88)=3.157 | .002^c^, *d*=.75 | 10.28(3.7) | 6.43(3.91) | *t*(85)= 4.401 | <.0001^c^, *d*=1.01 |
| Internalizing | 18.98(9.9) | 10.4(7.4) | *t*(175)=5.629 | <.0001^c^, *d*=.98 | 19.17(9.03) | 9.43(6.95) | *t*(88)=3.358 | <.0001^c^, *d*=1.21 | 18.83(10.7) | 11.52(7.79) | *t*(85)=3.271 | .002^c^, *d*=.78 |
| Externalizing | 13.21(5.28) | 8.54(5.04) | *t*(175)=5.535 | <.0001^c^, *d*=.90 | 13.13(6.19) | 8.19(5.16) | *t*(88)=3.214 | .0002^c^, *d*=.87 | 13.28(4.56) | 8.12(4.9) | *t*(85)=4.730 | <.0001^c^, *d*=.1.09 |
| Total problems | 75.00(22.91) | 52.19(18.21) | *t*(175)=7.017 | <.0001^c^, *d*=1.10 | 72.57(21.12) | 51.27(18.35) | *t*(88)=4.619 | <.0001^c^, *d*=.1.08 | 76.93(24.43) | 53.26(18.16) | *t*(85)=5.093 | <.0001^c^, *d*=1.10 |
|  |  |  |  |  |  |  |  |  |  |  |  |  |
| Middle: 14-16 years | *n*=97 | *n*=259 |  |  | *n*=40 | *n*=145 |  |  | *n*=57 | *n*=114 |  |  |
| Age: year, month | 15.18(.84) | 14.91(.79) | *t*(354)=2.831 | .005, *d*=.33 | 15.33(.84) | 14.97(.76) | *t*(183)=2.615 | .010, *d*=.47 | 15.07(.83) | 14.83(.83) | *t*(169)=1.806 | .073 |
| SES | 29.99(12.58) | 30.67(10.96) | *t*(354)=-.469 | .640 | 29.11(10.49) | 31.33(12.08) | *t*(183)=-1.043 | .299 | 30.59(13.89) | 29.82(9.3) | *t*(169)=.376 | .708 |
| Hb1Ac (%) | 8.24(1.4) | 7.66(1.16) | *t*(354)=3.965 | <.0001^c^, *d*=.45 | 8.16(1.21) | 7.62(1.17) | *t*(183)=2.589 | .010, *d*=.45 | 8.3(1.53) | 7.72(1.15) | *t*(169)=2.527 | .013, *d*=.43 |
| Duration of illness: year, month | 6.3(3.39) | 6.73(3.91) | *t*(354)=-1.036 | .301 | 6.97(3.85) | 6.84(3.92) | *t*(183)=.176 | .861 | 5.83(2.98) | 6.59(3.91) | *t*(169)=-1.425 | .156 |
| z-BMI | 1.12(1.28) | .38(.85) | *t*(354)=6.251 | <.0001^c^, *d*=.68 | 1.26(1.82) | .24(.89) | *t*(183)=4.973 | <.0001^c^, *d*=.71 | 1.02(.69) | .58(.74) | *t*(169)=3.724 | <.0001^c^, *d*=.61 |
| MDI/CSII/iport (*n*) | 65/29/2 | 143/113/3 | X^2^=5.446 | .066 | 30/10/0 | 79/64/2 | X^2^=.5662 | .059 | 35/19/2 ^a^ | 64/49/1 | X^2^=2.575 | .276 |
| Carb counting (yes) (*n*) ^b^ | 42 | 157 | X^2^=9.055 | .003, *V*=.160 | 16 | 90 | X^2^=6.241 | .012, *V*=.184 | 26 | 67 | X^2^=3.081 | .079 |
|  |  |  |  |  |  |  |  |  |  |  |  |  |
| YSR |  |  |  |  |  |  |  |  |  |  |  |  |
| Withdrawn | 5.08(3.15) | 3.36(2.66) | *t*(354)=4.783 | <.0001^c^, *d*=.59 | 3.48(2.37) | 3.09(2.46) | *t*(183)=.883 | .378 | 6.21(3.15) | 3.7(2.88) | *t*(169)=5.199 | <.0001^c^, *d*=.83 |
| Somatic complaints | 4.23(3.32) | 2.73(2.57) | *t*(354)=4.012 | <.0001^c^, *d*=.50 | 2.85(2.34) | 2.23(2.13) | *t*(183)=1.583 | .115 | 5.19(3.58) | 3.36(2.93) | *t*(169)=3.579 | <.0001^c^, *d*=.56 |
|  |  |  |  |  |  |  |  |  |  |  |  |  |
|  | Total sample | | | | Boys | | | | Girls | | | |
|  | DEBs  *n*=194 | No DEBs  *n*=496 | DEBs/no DEBs |  | DEBs  *n*=76 | No DEBs  *n*=277 | DEBs/no DEBs |  | DEBs  *n*=118 | No DEBs  *n*=219 | DEBs/no DEBs | DEBs  *n*=118 |
|  | *M(SD)* | *M(SD)* | *test* | *p, effect size* | *M(SD)* | *M(SD)* | *test* | *p, effect size* | *M(SD)* | *M(SD)* | *test* | *M(SD)* |
| Anxious/depressed | 8.34(5.65) | 5.06(3.42) | *t*(354)=5.368 | <.0001^c^, *d*=.70 | 4.78(3.6) | 4.32(3.02) | *t*(183)=.806 | .421 | 10.84(5.45) | 6.00(3.66) | *t*(169)=6.057 | <.0001^c^, *d*=1.04 |
| Social problems | 4.16(3.27) | 2.32(2.26) | *t*(354)=5.119 | <.0001^c^, *d*=.65 | 2.73(2.31) | 2.13(2.05) | *t*(183)=1.576 | .117 | 5.18(3.47) | 2.56(2.48) | *t*(169)=5.068 | <.0001^c^, *d*=.87 |
| Thought problems | 4.26(3.84) | 2.69(3.07) | *t*(354)=3.606 | <.0001^c^, *d*=.45 | 2.60(2.31) | 2.68(2.83) | *t*(183)=-.170 | .865 | 5.42(4.28) | 2.70(3.36) | *t*(169)=4.197 | <.0001^c^, *d*=.71 |
| Attention problems | 7.29(3.25) | 5.01(3.1) | *t*(354)=6.099 | <.0001^c^, *d*=.72 | 6.73(2.74) | 4.89(3.04) | *t*(183)=3.454 | .001^c^, *d*=.63 | 7.68(3.54) | 5.16(3.19) | *t*(169)=4.710 | <.0001^c^, *d*=.75 |
| Rule-breaking behavior | 3.84(3.15) | 2.47(2.36) | *t*(354)=3.891 | <.0001^c^, *d*=.49 | 4.10(1.91) | 2.71(2.49) | *t*(183)=3.009 | .003, *d*=.63 | 3.65(3.32) | 2.16(2.14) | *t*(169)=3.085 | .003, *d*=.53 |
| Aggressive behavior | 9.92(5.38) | 6.88(3.99) | *t*(354)=5.056 | <.0001^c^, *d*=.64 | 9.00(4.83) | 6.85(4.12) | *t*(183)=2.812 | .005, *d*=.48 | 10.56(5.69) | 6.93(3.83) | *t*(169)=4.352 | <.0001^c^, *d*=.75 |
| Internalizing | 17.65(10.82) | 11.15(7.1) | *t*(354)=5.494 | <.0001^c^, *d*=.71 | 11.1(7.05) | 9.64(6.11) | *t*(183)=1.291 | .198 | 22.25(10.67) | 13.06(7.8) | *t*(169)=5.773 | <.0001^c^, *d*=.98 |
| Externalizing | 13.75(7.7) | 9.35(5.66) | *t*(354)=5.141 | <.0001^c^, *d*=.64 | 13.1(7.06) | 9.56(5.96) | *t*(183)=3.192 | .002^c^, *d*=.54 | 14.21(8.13) | 9.09(5.26) | *t*(169)=4.326 | <.0001^c^, *d*=.75 |
| Total problems | 72.61(26.29) | 54.94(18.48) | *t*(354)=6.081 | <.0001^c^, *d*=.78 | 60.63(20.38) | 53.43(18.26) | *t*(183)=2.150 | .033, *d*=.37 | 81.02(26.85) | 56.85(18.67) | *t*(169)=6.098 | <.0001^c^, *d*=1.04 |
|  |  |  |  |  |  |  |  |  |  |  |  |  |
| Late: 17-19 years | *n*=45 | *n*=112 |  |  | *n*=13 | *n*=65 |  |  | *n*=32 | *n*=47 |  |  |
| Age: year, month | 17.44(.54) | 17.47(.62) | *t*(155)=-.243 | .808 | 17.45(.49) | 17.46(.58) | *t*(76)=-.065 | .949 | 17.44(.57) | 17.48(.67) | *t*(77)=-.274 | .785 |
| SES | 27.93(9.46) | 28.82(11.14) | *t*(155)=-.492 | .624 | 27.17(5.09) | 28.53(10.16) | *t*(76)=-.698 | .490 | 28.23(10.78) | 29.24(12.54) | *t*(77)=-.359 | .721 |
| Hb1Ac (%) | 8.43(1.35) | 7.32(1.11) | *t*(155)=5.276 | <.0001^c^, *d*=.90 | 7.87(.8) | 7.13(1.02) | *t*(76)=2.451 | .017, *d*=.81 | 8.65(1.47) | 7.57(1.21) | *t*(77)=3.563 | .001^c^, *d*=.80 |
| Duration of illness: year, month | 9.07(4.66) | 8.39(4.53) | *t*(155)=.842 | .401 | 7.98(5.25) | 8.56(4.79) | *t*(76)=-.933 | .695 | 9.51(4.42) | 8.16(4.17) | *t*(77)=1.382 | .171 |
| z-BMI | .74(1.05) | .15(.95) | *t*(155)=3.365 | .001^c^, *d*=.59 | .72(1.04) | -.04(1.05) | *t*(76)=2.360 | .021, *d*=.73 | .74(1.07) | .42(.73) | *t*(77)=1.634 | .106 |
| MDI/CSII/iport (*n*) | 28/17/0 | 58/54/0 | X^2^=1.412 | .235 | 9/4/0 | 40/25/0 | X^2^=.274 | .600 | 19/13/0 | 18/29/0 | X^2^=3.397 | .067 |
| Carb counting (yes) (*n*) ^b^ | 20 | 59 | X^2^=1.080 | .299 | 4 | 32 | X^2^=1.733 | .188 | 16 | 27 | X^2^=.426 | .514 |
|  |  |  |  |  |  |  |  |  |  |  |  |  |
|  |  |  |  |  |  |  |  |  |  |  |  |  |
|  | Total sample | | | | Boys | | | | Girls | | | |
|  | DEBs  *n*=194 | No DEBs  *n*=496 | DEBs/no DEBs |  | DEBs  *n*=76 | No DEBs  *n*=277 | DEBs/no DEBs |  | DEBs  *n*=118 | No DEBs  *n*=219 | DEBs/no DEBs | DEBs  *n*=118 |
|  | *M(SD)* | *M(SD)* | *test* | *p, effect size* | *M(SD)* | *M(SD)* | *test* | *p, effect size* | *M(SD)* | *M(SD)* | *test* | *M(SD)* |
| YSR |  |  |  |  |  |  |  |  |  |  |  |  |
| Withdrawn | 5.69(3.47) | 3.91(2.84) | *t*(155)=3.323 | .001^c^, *d*=.56 | 5.00(3.29) | 3.77(2.89) | *t*(76)=1.371 | .174 | 5.97(3.56) | 4.11(2.78) | *t*(77)=2.605 | .011, *d*=.58 |
| Somatic complaints | 4.78(2.94) | 2.71(2.23) | *t*(155)=4.244 | <.0001^c^, *d*=.80 | 3.23(1.42) | 2.34(2.16) | *t*(76)=1.425 | .158 | 5.41(3.17) | 3.23(2.26) | *t*(77)=3.341 | .002^c^, *d*=.79 |
| Anxious/depressed | 8.64(4.71) | 5.08(3.52) | *t*(155)=5.183 | <.0001^c^, *d*=.86 | 6.77(4.19) | 3.97(3.04) | *t*(76)=2.840 | .006, *d*=.76 | 9.41(4.76) | 6.62(3.6) | *t*(77)=2.966 | .004, *d*=.66 |
| Social problems | 4.13(3.36) | 2.73(2.38) | *t*(155)=2.551 | .013, *d*=.48 | 4.08(1.89) | 2.43(2.08) | *t*(76)=2.645 | .010, *d*=.83 | 4.16(3.83) | 3.15(2.72) | *t*(77)=1.369 | .175 |
| Thought problems | 4.76(4.44) | 2.34(2.26) | *t*(155)=3.472 | .001^c^, *d*=.69 | 4.23(3.47) | 2.11(2.16) | *t*(76)=2.896 | .005, *d*=.73 | 4.97(4.82) | 2.66(2.39) | *t*(77)=2.510 | .016, *d*=.61 |
| Attention problems | 7.20(3.47) | 5.05(2.78) | *t*(155)=3.701 | <.0001^c^, *d*=.68 | 8.15(3.62) | 5.09(2.93) | *t*(76)=3.304 | .001^c^, *d*=.93 | 6.81(3.83) | 5.00(2.6) | *t*(77)=2.560 | .013, *d*=.55 |
| Rule-breaking behavior | 4.00(3.01) | 3.02(2.3) | *t*(155)=1.971 | .053 | 5.31(2.78) | 3.29(2.3) | *t*(76)=2.782 | .007, *d*=.80 | 3.47(2.97) | 2.64(2.27) | *t*(77)=1.407 | .164 |
| Aggressive behavior | 9.4(5.03) | 7.19(3.85) | *t*(155)=2.655 | .010, *d*=.49 | 9.69(4.92) | 7.11(3.85) | *t*(76)=2.107 | .038, *d*=.58 | 9.28(5.14) | 7.30(3.9) | *t*(77)=1.948 | .05, *d*=.43 |
| Internalizing | 19.11(9.28) | 11.71(6.79) | *t*(155)=4.857 | <.0001^c^, *d*=.91 | 15.00(7.8) | 10.08(6.49) | *t*(76)=2.415 | .018, *d*=.68 | 20.78(9.42) | 13.96(6.62) | *t*(77)=3.546 | .001^c^, *d*=.84 |
| Externalizing | 13.40(7.38) | 10.21(5.57) | *t*(155)=2.620 | .011, *d*=.49 | 15.00(7.15) | 10.40(5.6) | *t*(76)=2.577 | .012, *d*=.72 | 12.75(7.48) | 9.94(5.56) | *t*(77)=1.971 | .059 |
| Total problems | 75.62(22.8) | 55.84(16.31) | *t*(155)=5.301 | <.001^c^, *d*=.99 | 72.85(16.25) | 53.2(16.61) | *t*(76)=3.907 | <.0001^c^, *d*=1.20 | 76.75(25.12) | 59.49(15.32) | *t*(77)=3.472 | .001^c^, *d*=.83 |

*Note*: Data are presented as mean values and standard deviation, unless otherwise stated; MDI=multiple day injection; CSII=continuous subcutaneous insulin infusion; iport= Injection port.

^a^ Missing data.

^b^ Answers indicating carb counting at least once per day.

^c^ Significant after Bonferroni’s correction (corrected alpha for accepting statistical significance: .05/18 tests = .0027).

**Table S3**

*Socio-demographic, clinical data, and YSR scores in adolescents with T1D with and without insulin misuse behaviors grouped by age*

|  | Early | | | |
| --- | --- | --- | --- | --- |
|  | Insulin misuse  *n*=78 | No insulin misuse  *n*=99 | Insulin misuse/no insulin misuse | |
|  | *M(SD)* | *M(SD)* | *test* | *p, effect size* |
|  |  |  |  |  |
| Age: year, month | 12.72(.66) | 12.72(.65) | *t*(175)=-.051 | .960 |
| SES | 29.76(11.58) | 29.2(9.18) | *t*(173)=.357 | .721 |
| Hb1Ac (%) | 7.98(1.23) | 7.79(1.92) | *t*(175)=1.074 | .284 |
| Duration of illness: year, month | 5.8(3.27) | 5.41(3.28) | *t*(175)=.785 | .433 |
| z-BMI | .43(.93) | .49(.92) | *t*(175)=-.449 | .654 |
| MDI/CSII/iport (*n*) | 46/31/1 | 66/31/1 | X^2^=1.316 | .518 |
| Carb counting (yes) (*n*)^a^ | 42 | 64 | X^2^=1.585 | .208 |
|  |  |  |  |  |
| Tot DEPS-r | 22.59(10.47) | 9.61(6.77) | *t*(175)=9.961 | <.0001^b^, *d*=1.47 |
|  |  |  |  |  |
| YSR |  |  |  |  |
| Withdrawn | 4.83(3.41) | 2.85(2.48) | *t*(175)=4.319 | <.0001^b^, *d*=.66 |
| Somatic complaints | 4.06(3.12) | 2.62(2.36) | *t*(175)=3.405 | .001^b^, *d*=.52 |
| Anxious/depressed | 7.36(4.88) | 4.83(3.89) | *t*(175)=3.739 | <.0001^b^, *d*=.57 |
| Social problems | 4.09(2.89) | 2.61(2.69) | *t*(175)=3.523 | .001^b^, *d*=.53 |
| Thought problems | 3.5(3.56) | 2.87(3.35) | *t*(175)=1.212 | .227 |
| Attention problems | 7.23(3.31) | 4.73(3.34) | *t*(175)=4.970 | <.0001^b^, *d*=.75 |
| Rule-breaking behavior | 2.94(1.98) | 1.91(1.97) | *t*(175)=3.428 | .001^b^, *d*=.52 |
| Aggressive behavior | 9.08(3.9) | 6.35(3.95) | *t*(175)=4.578 | <.0001^b^, *d*=.69 |
| Internalizing | 16.26(9.96) | 10.29(7.35) | *t*(175)=4.422 | <.0001^b^, *d*=.68 |
| Externalizing | 12.01(5.03) | 8.26(5.35) | *t*(175)=4.751 | <.0001^b^, *d*=.72 |
| Total problems | 67.7(22.15) | 52.03(19.86) | *t*(175)=4.922 | <.0001^b^, *d*=.74 |

|  | Middle | | | | Late | | | |
| --- | --- | --- | --- | --- | --- | --- | --- | --- |
|  | Insulin misuse  *n*=131 | No insulin misuse  *n*=225 | Insulin misuse/no insulin misuse | | Insulin misuse  *n*=60 | No insulin misuse  *n*=97 | Insulin misuse/no insulin misuse | |
|  | *M(SD)* | *M(SD)* | *test* | *p, effect size* | *M(SD)* | *M(SD)* | *test* | *p, effect size* |
|  |  |  |  |  |  |  |  |  |
| Age: year, month | 14.98(.85) | 14.98(.8) | *t*(353)=-.040 | .968 | 17.5(.65) | 17.43(.56) | *t*(155)=.829 | .408 |
| SES | 29.77(11.06) | 30.97(11.59) | *t*(348)=-.948 | ~~.030,~~ *~~d~~*~~=.23~~ .344 | 28.67(9.63) | 28.5(11.34) | *t*(145)=.095 | .924 |
| Hb1Ac (%) | 8.00(1.33) | 7.71(1.96) | *t*(353)=2.138 | .033, *d*=.17 | 7.97(1.36) | 7.43(1.98) | *t*(155)=2.597 | .010, *d*=.32 |
| Duration of illness: year, month | 6.56(3.76) | 6.62(3.77) | *t*(353)=-.151 | .880 | 8.53(4.58) | 8.62(4.58) | *t*(155)=-.125 | .~~041,~~ *~~d~~*~~=.33~~ .901 |
| z-BMI | .69(1.3) | .52(.84) | *t*(353)=1.485 | .138 | .25(1.09) | .37(.97) | *t*(155)=-.714 | .476 |
| MDI/CSII/iport (*n*) | 106/70/5 | 101/72/0 | X^2^=4.971 | .083 | 42/34 | 44/37 | X^2^=.014 | .906 |
| Carb counting (yes) (*n*)^a^ | 70 | 128 | X^2^=.421 | .516 | 22 | 57 | X^2^=7.133 | .008, *V*=.215 |
|  |  |  |  |  |  |  |  |  |
| Tot DEPS-r | 22.47(12.19) | 11.64(7.91) | *t*(353)=9.078 | <.0001^b^, *d*=.1.05 | 20.92(12.77) | 12.13(9.26) | *t*(155)=4.628 | <.0001^b^, *d*=.79 |
|  |  |  |  |  |  |  |  |  |
| YSR |  |  |  |  |  |  |  |  |
| Withdrawn | 4.32(2.78) | 3.54(2.95) | *t*(353)=2.468 | .014, *d*=.27 | 5.08(3.3) | 4.01(2.96) | *t*(155)=2.112 | .036, *d*=.34 |
| Somatic complaints | 3.48(2.77) | 2.93(2.91) | *t*(353)=-1.748 | .081 | 3.63(2.88) | 3.10(2.44) | *t*(155)=1.234 | .219 |
| Anxious/depressed | 6.68(4.58) | 5.52(4.23) | *t*(353)=2.414 | .016, *d*=.26 | 6.87(4.7) | 5.63(3.82) | *t*(155)=1.805 | .073 |
| Social problems | 3.46(2.85) | 2.44(2.53) | *t*(353)=3.494 | .001^b^, *d*=.38 | 3.73(2.76) | 2.76(2.71) | *t*(155)=2.164 | .032, *d*=.35 |
| Thought problems | 3.79(3.25) | 2.73(3.39) | *t*(353)=2.892 | .004, *d*=.32 | 3.52(3.85) | 2.73(2.76) | *t*(155)=1.486 | .139 |
| Attention problems | 6.58(3.25) | 5.08(3.21) | *t*(353)=4.233 | <.0001^b^, *d*=.46 | 6.62(3.46) | 5.08(2.78) | *t*(155)=2.901 | .005, *d*=.49 |
| Rule-breaking behavior | 3.54(2.89) | 2.44(2.44) | *t*(353)=3.646 | <.0001^b^, *d*=.41 | 3.85(2.59) | 2.96(2.48) | *t*(155)=2.150 | .033, *d*=.35 |
| Aggressive behavior | 8.68(4.68) | 7.15(4.49) | *t*(353)=3.037 | .003, *d*=.33 | 8.23(4.63) | 7.57(4.13) | *t*(155)=.938 | .350 |
| Internalizing | 14.49(8.69) | 12.00(8.7) | *t*(353)=2.606 | .010, *d*=.29 | 15.58(9.06) | 12.74(7.59) | *t*(155)=2.115 | .036, *d*=.34 |
| Externalizing | 12.22(6.87) | 9.59(6.21) | *t*(353)=3.688 | <.0001^b^, *d*=.4 | 12.08(6.64) | 10.53(6.01) | *t*(155)=1.515 | .132 |
| Total problems | 65.65(22.68) | 56.31(21.43) | *t*(353)=3.873 | <.0001^b^, *d*=.42 | 66.52(22.84) | 58.41(18.18) | *t*(155)=2.457 | .015, *d*=.39 |

*Note:* Data are presented as mean values and standard deviation, unless otherwise stated; MDI=multiple day injection; CSII=continuous subcutaneous insulin infusion; iport= Injection port

^a^ Answers indicating carb counting at least once per day.

^b^ Significant after Bonferroni’s correction (corrected alpha for accepting statistical significance: .05/19 tests =.0026).
